# Impact of Social Determinants of Health on the Incidence of Tuberculosis in Central Asia

**DOI:** 10.3390/ijerph23010068

**Published:** 2026-01-01

**Authors:** Assiya Kussainova, Laura Kassym, Almas Kussainov, Ainash Orazalina, Yerbol Smail, Gulmira Derbissalina, Zhanagul Bekbergenova, Ulzhan Kozhakhmetova, Elvira Aitenova, Yuliya Semenova

**Affiliations:** 1 Department of General Medical Practice with a Course of Evidence-Based Medicine, Astana Medical University, Astana 010000, Kazakhstan; kussainova.as@amu.kz (A.K.); derbissalina.g@amu.kz (G.D.); bekbergenova.zh@amu.kz (Z.B.); kozhakhmetova.u@amu.kz (U.K.); elya_amu_89@mail.ru (E.A.); 2 Department of Psychiatry and Narcology, NJSC Astana Medical University, Astana 010000, Kazakhstan; 3 Department of Molecular Biology and Medical Genetics Named After the Academician of National Academy of Sciences Republic of Kazakhstan Raissov T. K., NJSC Semey Medical University, Semey 071400, Kazakhstan; ainash.orazalina@smu.edu.kz; 4 Department of Infectious Diseases, Dermatovenereology and Immunology, NJSC Semey Medical University, Semey 071400, Kazakhstan; erbol.smail@smu.edu.kz; 5 School of Medicine, Nazarbayev University, Astana 010000, Kazakhstan; yuliya.semenova@nu.edu.kz

**Keywords:** tuberculosis, incidence, social determinants of health, Kazakhstan, Central Asia

## Abstract

Background/Objectives: Tuberculosis (TB) remains a major global health challenge influenced by social determinants of health (SDHs) such as poverty, overcrowding, malnutrition, and limited healthcare access. Although Central Asia (CA) has achieved progress through vaccination, screening, and treatment, the region continues to face severe disease consequences, unstable incidence patterns, and an escalating challenge of TB resistant to first-line drugs. This study aimed to analyze TB incidence dynamics in Kazakhstan, Tajikistan, the Kyrgyz Republic, Turkmenistan, and Uzbekistan from 2000–2023, forecast trends to 2030, and identify key SDHs shaping the epidemic. Methods: Data on TB incidence were obtained from the World Bank DataBank for 2000–2023. Of 61 socioeconomic, environmental, and health-related indicators, 29 were included in the analysis. Statistical procedures in SPSS (v24.0) involved time-series forecasting through 2030, calculation of average annual percentage change (AAPC), correlation testing, and linear regression, with significance set at *p* < 0.05. Results: TB incidence generally declined across CA during 2000–2023, though trends varied by country. Forecasts suggest continued decreases in Turkmenistan and Uzbekistan, while Kazakhstan, Tajikistan, and the Kyrgyz Republic display fluctuating or nonsignificant patterns, likely influenced by SDHs. Regression analyses indicated that anemia, undernourishment, and population density showed a positive relationship with TB incidence, while clean fuel access, physician density, and Gross Domestic Product per capita (GDP) were inversely related. Conclusions: The findings highlight the heterogeneous nature of TB dynamics in CA and the possible role of SDHs. Enhanced surveillance, nutritional and social interventions are required to sustain progress toward End TB targets.

## 1. Introduction

Tuberculosis (TB) remains one of the most prevalent infectious diseases worldwide and is caused by *Mycobacterium tuberculosis* (*MTB*) [1]. According to estimates from the World Health Organization (WHO), approximately 2.5 billion individuals are infected with latent tuberculosis, reflecting an enormous hidden epidemiological burden and a persistent threat to nearly one-third of the global population [2,3]. International initiatives play a decisive role in addressing this challenge, notably the WHO “End TB Strategy” and the United Nations Sustainable Development Goals (SDGs), both of which aim to reduce incidence and mitigate the broader socioeconomic impact of the disease. The 2025 TB strategy set the goal of achieving a 50% reduction in incidence compared with baseline levels [4,5]. However, actual progress has fallen considerably short of these benchmarks. While in high-income countries such as the United States, Canada, and most European states, TB incidence rarely surpasses 10 cases per 100,000 people, the situation remains critical in low- and middle-income regions, particularly in South and Southeast Asia, where the disease continues to impose a disproportionately high burden [5].

The incidence of TB is strongly shaped, both positively and negatively, by a wide range of socioeconomic determinants, including demographic, environmental, and behavioral factors [6]. Since the WHO formally endorsed the concept of social determinants of health (SDHs) in 2008, it has emphasized that the unequal distribution of these determinants represents one of the primary drivers of disparities in morbidity [7]. Nguipdop-Djomo P. et al. (2020) reported that low living standards, overcrowded housing, limited access to health services, homelessness, and tobacco smoking substantially increase the risk of developing TB [8]. Additional contributors include malnutrition and comorbid conditions, which not only compromise therapeutic outcomes but also facilitate the emergence of drug resistance [9]. The use of solid fuels for cooking and heating further exacerbates this problem by releasing harmful emissions that damage pulmonary tissues and increase susceptibility to infection [10]. Moreover, the specific configuration of determinants shaping TB transmission varies across countries, underscoring the need for context-specific investigations and mitigation strategies.

In recent years, countries in Central Asia (CA) have demonstrated a notable decline in TB incidence [11]. The widespread implementation of Bacillus Calmette–Guérin (BCG) vaccination programs, large-scale screening initiatives, chemotherapy, and the adoption of the Directly Observed Treatment, Short-course (DOTS) strategy have contributed to substantial progress in TB control [12]. For example, an analysis of Kazakhstan’s Unified National Electronic Health System revealed that between 2014 and 2019, the incidence rate fell from 227.0 to 69.1 per 100,000 people [13]. Similarly, data from the Agency on Statistics under the President of the Republic of Tajikistan (2018) reported a reduction in TB incidence from 220 cases in 2002 to 78 cases in 2022 [14]. In the Kyrgyz Republic, the incidence decreased from 165–144 cases per 100,000 population over the decade from 2007–2017 [15]. Comparable patterns were also observed in Turkmenistan and Uzbekistan [16]. However, the absence of comprehensive studies covering all CA countries, the fragmented and chronologically inconsistent nature of existing research, and the lack of analyses forecasting future TB trends collectively highlight the need for more systematic and comparable investigations.

Despite encouraging trends, researchers continue to report fluctuations in TB incidence driven by various risk factors [13,15,16]. The impact of the COVID-19 pandemic should also be acknowledged, as it may have caused substantial disruptions in case detection, delays in diagnostic services, and interruptions in routine TB surveillance [17]. All countries in the region have introduced National Action Plans (NAPs) aimed at curbing the rise of TB, although the effectiveness of these measures varies considerably across settings [13]. In addition, TB incidence data are regularly reported to the WHO Regional Office for Europe [18] and supplemented by independent studies; however, the contribution of SDHs to TB epidemiology in this region has not been systematically assessed. Only a few studies have examined SDHs within Kazakhstan, and these analyses have typically been limited to specific provinces rather than the country as a whole [19,20]. For example, Sorokina M. et al. (2023) demonstrated that economic development and healthcare capacity are inversely correlated with TB incidence [19]. Given the importance of these determinants, their systematic assessment could serve as a foundation for designing more effective policy measures and targeted intervention strategies.

Accordingly, the aims of this study are (i) to analyze the dynamics of TB incidence in Kazakhstan, Tajikistan, the Kyrgyz Republic, Turkmenistan, and Uzbekistan between 2000 and 2023; (ii) to forecast epidemiological trends through 2030; and (iii) to identify the key SDHs of health that influence disease patterns in the region.

## 2. Materials and Methods

### 2.1. Countries Examined

**Figure 1:**
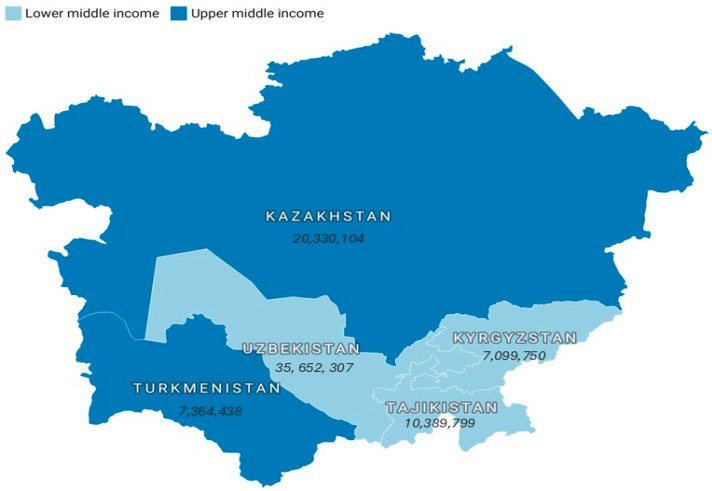
Cartographic presentation of the study sample, highlighting World Bank income classifications alongside population data for 2023.

CA is a landlocked region of Eurasia comprising Kazakhstan, Tajikistan, the Kyrgyz Republic, Turkmenistan, and Uzbekistan. The World Bank (WB) classifies CA states as upper-middle-income (Kazakhstan and Turkmenistan) and lower-middle-income (Kyrgyz Republic, Uzbekistan, and Tajikistan). The database contains data on TB incidence across all five countries for the period 2000–2023. Figure 1 displays a map of the countries examined, highlighting their WB income classifications and 2023 population sizes [21].

### 2.2. Data Origins

Data for the study were chiefly obtained from the World Bank DataBank (WB DataBank). The WB DataBank is an online analysis and visualization tool that provides access to a wide range of time series datasets on global economic, social, and environmental indicators, enabling users to query, compare, and download data for research and policy purposes [21].

### 2.3. Variables Investigated

This study employs a time-trend correlation analysis conducted separately for each country to examine associations between annual TB incidence and SDHs. TB incidence, expressed per 100,000 people, was designated the primary study variable and served as the outcome measure. According to the definition of the WB DataBank, the incidence of TB is the estimated number of new and relapsing TB cases arising in a given year, expressed as the rate per 100,000 people [21]. Building on prior research that has identified a range of health, social, and environmental determinants [22,23,24], we selected predictor variables spanning multiple domains, including air pollution, health-related behaviors, economic development and equity, education, employment, healthcare access and services, health expenditure, and population characteristics. These variables were obtained primarily from the WB DataBank [21] and subsequently subjected to a systematic assessment of their appropriateness for analysis. Variables were excluded primarily on the basis of incomplete data availability, particularly when records did not cover the full timeframe of 2000–2023. From the original set of 61 variables, 29 fulfilled the inclusion requirements and were incorporated into the study analysis. The Supplementary Materials present a detailed list of both included and excluded variables, together with the rationale for exclusion (Supplementary Tables S1 and S2).

### 2.4. Data Analysis

The extracted variables were systematically arranged in Excel spreadsheets and included as Supplementary Materials. Country-specific spreadsheets documenting TB incidence were developed to assess longitudinal trends, whereas additional spreadsheets for predictors and outcomes were organized to investigate potential associations. The Statistical Package for the Social Sciences (IBM SPSS Statistics for Windows, version 24.0; IBM Corp., Armonk, NY, USA) was used for all analyses, with statistical significance established at a 5% Type I error level (*p* < 0.05).

Historical trends across the study period were assessed via time series methods, and forecasts were generated through 2030. The average annual percentage change (AAPC) with 95% confidence intervals was used to quantify past changes. For prediction, the Expert Modeler procedure in SPSS identified the best-fitting model. The forecasted values, corresponding graphs, and statistical characteristics of the selected model, including its *p*-value, are presented in the report. Model adequacy was evaluated using residual diagnostics, including inspection of ACF/PACF plots and the Ljung–Box test to confirm the absence of residual autocorrelation. Model fit was assessed using AIC/BIC. SPSS Expert Modeler selected the optimal forecasting approach for each country based on the statistical characteristics of its time series. Autoregressive Integrated Moving Average (ARIMA) models were chosen for series that became stationary after differencing, whereas Holt exponential smoothing was selected for series with persistent trends and no clear autoregressive structure. The use of different model types does not affect comparability, as forecasts reflect the best-fitting model for each country rather than differences in modelling strategy. All reported *p*-values reflect the global F-test, indicating overall model significance.

The analysis of the associations between predictors and TB incidence was performed in a sequential manner. As a first step, the analysis included only those predictor variables with missing values not exceeding 10%, defined as at most one missing observation per variable. The mean of the nonmissing time points was used to impute missing values. Pearson’s correlation analysis was applied to all the study variables to assess potential multicollinearity. Correlations used to assess multicollinearity (r ≥ 0.8) were calculated within each country across the 24-year time series, allowing us to identify predictors that moved in parallel over time. All 29 candidate variables were initially screened; however, the final set of predictors was informed by both empirical correlations and theoretical relevance based on prior literature. When pairs of highly correlated variables were identified, we retained the variable with the strongest association with TB incidence and clearer conceptual justification (Supplementary Table S3). Linear regression analyses were subsequently carried out via both the enter approach and the backward elimination procedure. Through backward elimination, variables that fail to meet the significance criterion (*p* < 0.05) are iteratively excluded until only significant predictors remain in the final regression model. Standardized beta estimates accompanied by 95% CI lower and upper bounds were reported. The coefficient of determination (R^2^) was used to evaluate the model’s predictive ability. The levels of significance were classified as statistically significant (*p* < 0.05), highly significant (*p* < 0.01), or very highly significant (*p* < 0.001).

### 2.5. Ethics Statement

The Ethics Committee of Astana Medical University approved this study (Approval No. 13; 27 September 2024) in accordance with the principles of the 1964 Declaration of Helsinki. Although only publicly available aggregated data were used, ethics approval was obtained in line with institutional policy requiring review of all research activities.

## 3. Results

### 3.1. The Observed and Projected TB Incidence Rates in Central Asian Countries

**Figure 2:**
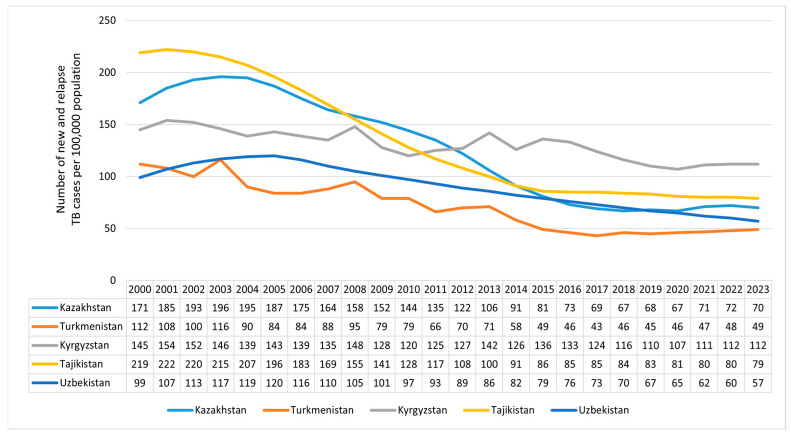
Incidence of tuberculosis in Central Asia (2000–2023).

Figure 2 presents trends in the number of new and relapsing TB cases per 100,000 population from 2000–2023 across five CA countries: Kazakhstan, the Kyrgyz Republic, Tajikistan, Turkmenistan, and Uzbekistan. All five countries demonstrated a general decline in TB incidence rates over the 24-year period.

Kazakhstan demonstrated a substantial and consistent decline in TB incidence, decreasing from 171 cases per 100,000 population in 2000 to 70 cases in 2023, representing the largest reduction among the five countries (AAPC: −5.60%, *p* < 0.001). In Kyrgyzstan, incidence declined modestly from 145 to 112 cases per 100,000 over the same period; however, the trend showed a slightly increasing AAPC (1.37%, *p* < 0.001). Tajikistan exhibited a pronounced reduction, with incidence falling from 219 to 79 cases per 100,000 (AAPC: −5.38%, *p* < 0.001). Turkmenistan similarly experienced a marked decline, from 112 to 49 cases per 100,000 (AAPC: −4.42%, *p* < 0.001). Uzbekistan also showed a steady decrease, with incidence dropping from 99 to 57 cases per 100,000 (AAPC: −3.15%, *p* < 0.001).

Table 1 summarizes the projected TB incidence trends from 2024–2030 in five CA countries. In Kazakhstan, ARIMA modelling (1.2.0) suggests a steady year-on-year decrease from 66 cases per 100,000 population in 2024 to 38 cases by 2030 (*p =* 0.015). Kyrgyzstan, modeled with Holt exponential smoothing, is projected to show a more gradual decline, with incidence decreasing from 108 to 98 cases per 100,000 over the same period (*p* = 0.487). For Tajikistan, the ARIMA (0.3.0) model indicates a consistent downward trend from 77 cases in 2024 to 60 cases in 2030 (*p* = 0.623). Turkmenistan, modeled using the Holt method, is projected to experience one of the sharpest declines, from 42 cases in 2024 to 23 cases in 2030 (*p* = 0.020). Uzbekistan is also expected to continue its downward trajectory, with ARIMA (0.2.0) forecasts showing a reduction from 54 cases per 100,000 in 2024 to 22 cases in 2030 (*p* = 0.126).

### 3.2. SDHs of TB Incidence in Countries in Central Asia, 2000–2023

Table 2 presents the results of multivariable regression analyses examining the associations between TB incidence and selected predictors in Kazakhstan. Access to clean cooking fuels showed a strong and highly significant negative association with TB incidence in both models (*p* < 0.001). A greater proportion of children aged 0–14 years was also significantly associated with lower TB rates (*p* < 0.001). Population density demonstrated a positive and significant relationship with TB incidence, especially in Model 2 (*p* < 0.001). The prevalence of undernourishment was marginally significant in Model 1 (*p* = 0.047) but not in Model 2. Life expectancy at birth showed no significant association and was excluded from the final model.

Table 3 summarizes the findings of multivariable regression analyses assessing the relationships between TB incidence and several predictors in the Kyrgyz Republic. The prevalence of anemia among nonpregnant women showed a strong positive association with TB incidence, reaching high statistical significance in Model 2 (*p* < 0.001). Life expectancy at birth was not significantly associated with TB incidence and was excluded from the final model.

Table 4 displays the outcomes of multivariable regression analyses assessing the relationships between TB incidence and various SDHs in Tajikistan over the period 2000–2023. GDP per capita showed a strong and highly significant negative association with TB incidence in both models (*p* < 0.001). Population density was also negatively associated with TB incidence (*p* < 0.05). The prevalence of anemia among nonpregnant women was strongly positively associated with TB incidence (*p* < 0.01). Undernourishment was similarly linked to higher TB rates, with significant positive coefficients in both models. Measles immunization was not significantly associated with TB and was excluded from the final model.

Table 5 outlines the findings of multivariable regression analyses evaluating the associations between TB incidence and several SDHs in Turkmenistan. Physician density showed a strong and statistically significant negative association with TB incidence in both models. The prevalence of anemia among nonpregnant women demonstrated a very strong and highly significant positive relationship with TB incidence. Greenhouse gas emissions showed marginal significance in Model 1 (*p* = 0.056) and were excluded from Model 2, indicating a weak and inconsistent association.

Table 6 presents multivariable regression analyses examining associations between SDHs and TB incidence in Uzbekistan. Life expectancy at birth showed a strong and statistically significant negative association with TB incidence in Model 1 and Model 2 (*p* = 0.027 and *p* < 0.001, respectively). In contrast, current health expenditure per capita (*p* = 0.652) and GDP per capita (*p* = 0.503) did not have significant effects. Additionally, current health expenditure per capita and GDP per capita were not statistically significant and were removed from the final model. Although the explained variance was slightly lower (R^2^ = 0.780), the difference from Model 1 was not statistically significant (*p* = 0.213).

## 4. Discussion

### 4.1. Overall Trends in TB Incidence in Central Asia and Beyond the Study Areas

This study examined long-term trends in TB incidence across five CA countries over 2000–2023, generated forecasts through 2030, and assessed the associations between the key SDHs and TB rates. The results indicate a substantial decline in TB incidence throughout the region over the past 24 years, though the pace and continuity of this reduction varied across countries and time periods. In Kazakhstan and Tajikistan, the sharpest decrease occurred between 2005 and 2015, likely reflecting the expansion of national TB control programs, intensified implementation of DOTS/”End TB Strategy” measures [4], strengthened infection-control policies, increased health sector financing, and a gradual shift toward primary care–based diagnostic and treatment models [25]. After 2015, however, the concentration of cases in socially vulnerable groups and a rising proportion of drug-resistant forms appear to have contributed to a slowdown in progress [14,26,27]. In contrast, the fluctuations observed in the Kyrgyz Republic and Turkmenistan between 2006 and 2015 may be linked to interruptions in antituberculosis drug supply chains, a substantial burden of drug-resistant TB, and significant labor migration. These alternating periods of decline and resurgence likely increased treatment discontinuation and perpetuated transmission [28]. Unlike its regional counterparts, Uzbekistan demonstrated a consistent decline in TB incidence, reportedly associated with reductions in treatment failure rates and the introduction of new therapeutic regimens [16].

Beyond the study area, TB epidemiology in other post-Soviet countries also demonstrates considerable heterogeneity [29,30,31,32]. In the Russian Federation, substantial regional disparities in TB incidence persist, largely driven by socioeconomic inequalities and uneven access to healthcare services [11]. Particularly concerning are the challenging conditions within the penitentiary system, where limited availability of medical care contributes to delayed diagnosis and continued transmission of the disease [29]. Belarus has experienced a notable long-term decline in TB incidence—from 86 to 28 per 100,000 population between 2000 and 2022—yet continues to report a high burden of drug-resistant TB forms, with resistant strains detected in 35.3% of newly diagnosed cases and 76.5% of previously treated cases [30,31]. Ukraine has demonstrated rising TB incidence amid armed conflict, especially in central regions [32]. Comparable patterns of epidemiological variability are observed across several low- and middle-income countries, including India, Pakistan, and the Philippines, where socioeconomic disparities and uneven healthcare access sustain high transmission levels [33,34,35].

### 4.2. Social Determinants of Health and TB Incidence

Among the most relevant SDHs identified as possible contributors to TB incidence in this study are demographic factors such as population density, the proportion of children aged 0–14 years, and life expectancy at birth. In Kazakhstan, population density demonstrated a strong and statistically significant positive association with TB incidence (*p* < 0.001). This observation aligns with earlier evidence showing that overcrowded environments increase exposure risk and facilitate community transmission [36]. High population concentration in low- and middle-income settings also tends to amplify structural vulnerabilities, including inadequate sanitation, substandard housing, congested transport, and limited health resources, which together create conditions conducive to TB persistence [36]. By contrast, Tajikistan exhibited a statistically significant negative association between population density and TB incidence (*p* < 0.05). This pattern may reflect urbanization dynamics in which more densely populated urban centers benefit from better healthcare access, earlier detection, and timely initiation of treatment, thereby lowering TB incidence [37,38].

Similarly, the proportion of children aged 0–14 years demonstrated an inverse association with TB incidence in Kazakhstan (*p* < 0.001). This finding is consistent with the fact that adults constitute the main source of TB transmission, while high BCG coverage, regular preventive examinations, and strong pediatric follow-up systems contribute to lower incidence in this age group [39,40].

Life expectancy at birth did not exhibit a significant association with TB incidence in Kazakhstan, whereas in Uzbekistan, a strong negative relationship was identified (*p* < 0.001), consistent with life expectancy functioning as an integrative indicator of societal development and healthcare system performance [41].

Of the economic determinants assessed, GDP per capita showed a statistically significant negative association with TB incidence only in Tajikistan (*p* < 0.001). As a lower-middle-income country, even modest economic improvements translate into better nutrition, living conditions, and access to health services—all factors that contribute to reducing TB risk [21]. This result is in line with broader international evidence demonstrating that higher GDP per capita is associated with lower TB incidence and related health burden [42,43].

Access to clean fuels, reflected in reductions in exposure to greenhouse gas emissions, was negatively associated with TB incidence in Kazakhstan (*p* < 0.001). Smoke from solid fuels is known to damage respiratory mucosa and increase susceptibility to TB infection [44]. Kazakhstan’s ongoing transition away from solid fuels toward expanded gas infrastructure is therefore likely to yield public health benefits, with projections suggesting that by 2030, approximately 97% of the population will have access to clean fuels [45]. Evidence indicates that eliminating tobacco use and solid fuel exposure together could reduce TB incidence by 14–27% depending on DOTS coverage levels [44].

Economic stability also appears to influence TB control through its effect on healthcare workforce availability. In Turkmenistan, a negative association between TB incidence and the number of healthcare personnel likely reflects improved access to services, more consistent preventive activities, and more effective monitoring of treatment adherence [46].

The high prevalence of anemia among nonpregnant women in the Kyrgyz Republic, Tajikistan, and Turkmenistan, together with the observed associations between undernutrition and TB incidence in Kazakhstan and Tajikistan, reflects broader socioeconomic vulnerability. Rather than indicating a direct biological causal pathway, these associations likely stem from underlying resource deprivation, including limited access to adequate nutrition and essential health services. Although anemia and undernutrition may impair immune function, in this context, they primarily serve as proxy indicators of structural disadvantages such as food insecurity and constrained healthcare access [47,48,49,50].

### 4.3. Implications for Public Health Policy

The findings of this study have direct implications for strengthening TB control policies across CA countries. Given the projected heterogeneity in TB incidence trajectories for 2024–2030 and the differing influence of SDHs, country-specific and sector-integrated approaches are required rather than a single regional strategy [51]. In Kazakhstan, where declining reliance on solid fuels is associated with lower TB incidence, TB programs could benefit from closer collaboration with the energy and housing sectors to accelerate clean-fuel adoption, improve ventilation standards, and reduce exposure to biomass smoke, interventions supported by global evidence linking household air pollution to increased TB risk [52,53]. Strengthening regulatory frameworks for urban housing quality may further mitigate transmission in densely populated urban settings.

The consistent association between anemia and TB incidence in the Kyrgyz Republic, Tajikistan, and Turkmenistan underlines the need for integrating nutritional support and targeted anemia control into national TB programs. Evidence from randomized and observational studies suggests that iron deficiency and undernutrition substantially increase susceptibility to TB infection and progression [54,55]. Accordingly, policy actions may include routine nutritional assessment of TB-affected households, micronutrient supplementation for women of reproductive age, and social protection measures such as food baskets or cash transfers, strategies that align with WHO recommendations for TB-affected populations [56].

Similarly, the documented association between undernourishment and higher TB incidence in Kazakhstan and Tajikistan highlights the value of integrating food security programs into national TB strategies. Global modelling studies estimate that reducing undernutrition could avert up to 38% of global TB cases [56], making nutritional interventions a high-impact policy instrument. These findings directly support the inclusion of nutrition-sensitive actions within NAPs and align with SDGs-2 and SDGs-3 priorities.

Beyond disease-specific programming, the study’s results can inform broader multisectoral interventions. For example, findings regarding healthcare workforce availability and urbanization patterns can be incorporated into revisions of NAPs, particularly for improving early detection, enhancing treatment adherence support, and adapting service delivery models for migratory or socially vulnerable groups. Strengthening coordination between TB programs and ministries responsible for labor migration, social protection, and housing will be essential—especially given the documented role of labor mobility and socioeconomic instability in sustaining TB transmission in the region [11].

### 4.4. Strengths and Limitations

This study represents the first comprehensive multicountry analysis of TB incidence trends across all CA states between 2000 and 2023, integrating a wide range of SDHs. The use of long-term national datasets provided valuable insights into patterns that may support evidence-informed policy development within the region. The inclusion of multiple determinants allowed for the examination of broader structural processes shaping TB risk, thereby contributing to a more holistic understanding of TB epidemiology in CA.

Despite these strengths, several important limitations should be acknowledged. First, the study is ecological in design and relies on country-level aggregate data. As such, the models cannot establish causality, and all associations must be interpreted as exploratory rather than confirmatory. Because ecological analyses cannot resolve individual-level mechanisms, the risk of ecological fallacy remains inherent, and no inferences can be made about the risk profiles of specific population groups. Furthermore, several relevant variables, such as migration flows, the proportion of the population living in poverty, and tobacco-use prevalence, could not be incorporated due to limited data availability, which may introduce unmeasured confounding.

Second, the relatively small number of annual observations per country (*n* ≈ 24) combined with the inclusion of multiple predictors increases the likelihood of model overfitting. This issue should be considered when interpreting the regression coefficients and projected trends. Although mean imputation was applied for variables with ≤10% missing data, the lack of a formal sensitivity analysis represents an additional limitation and may affect the robustness of the estimates. Future studies using more advanced imputation or time-series interpolation methods could help evaluate the stability of these findings.

Third, several constraints related to data structure limited the ability to assess the impact of emerging factors such as the COVID-19 pandemic. Specifically, in the absence of quarterly or monthly TB incidence data, it was not possible to quantify pandemic-related disruptions in diagnosis or case detection, as documented in other international studies. Likewise, the available databases did not include indicators needed to examine latent tuberculosis infection, preventing an assessment of COVID-19–related risks of TB reactivation.

Finally, the completeness of reporting and variations in national surveillance methodologies may introduce additional sources of bias and affect comparability across countries. Despite these limitations, the findings provide meaningful region-specific insights and highlight key socioeconomic conditions that warrant further investigation using more granular and longitudinal data.

## 5. Conclusions

This study demonstrates that SDHs play a pivotal role in shaping the incidence and distribution of TB across CA. Persistent disparities in income, employment, housing, and healthcare access contribute to regional differences and sustain transmission despite overall epidemiological improvements. Achieving sustainable reductions in TB burden therefore requires integrating social protection, nutritional support, and community-based interventions with existing medical programs. Strengthened surveillance, targeted drug-resistant TB control, and multisectoral collaboration are essential to sustain progress toward the WHO End TB Strategy goals.

## Figures and Tables

**Table 1:** Projected tuberculosis incidence rates in Central Asian countries, expressed as new and relapsed cases per 100,000 people until 2030.

Year	Country				
	Kazakhstan	Kyrgyzstan	Tajikistan	Turkmenistan	Uzbekistan
2024	66	108	77	42	54
2025	62	106	75	39	50
2026	58	105	72	35	45
2027	53	103	69	32	40
2028	48	101	66	29	35
2029	43	99	63	26	29
2030	38	98	60	23	22
Model parameters	ARIMA (1.2.0) *p* = 0.015 ^+^	Holt *p* = 0.487	ARIMA (0.3.0) *p* = 0.623	Holt *p* = 0.020 ^+^	ARIMA (0.2.0) *p* = 0.126

*^+^ The association was statistically significant.*

**Table 2:** Multivariable associations between the incidence of TB and predictors in Kazakhstan, (*n* = 24 observations per country, historical period: 2000–2023).

Predictors	Model 1 (Enter Method)		Model 2 (Backward Method)	
	Standardized Coefficients (95% CI (Lower; Upper))	*p*- Value	Standardized Coefficients (95% CI (Lower; Upper))	*p*- Value
Access to clean fuels and technologies for cooking	−0.644 (−16.779; −5.725)	<0.001 °	−0.777 (−16.222; −10.922)	<0.001 °
Life expectancy at birth, total	−0.103 (−4.541; 1.660)	0.328	-	-
Population ages 0–14	−0.638 (−24.859; −11.706)	<0.001 °	−0.733 (−24.338; −17.639)	<0.001 °
Population density	0.298 (3.356; 52.930)	0.028 *	0.386 (18.961; 54.039)	<0.001 °
Prevalence of undernourishment	0.098 (0.042; 5.314)	0.047 *	0.085 (−0.199; 4.837)	0.069
Model parameters	R^2^ = 0.991; overall model *p* < 0.001 °		R^2^ = 0.991; overall model *p* = 0.328	

* The association was highly significant. ° The association was statistically highly significant.

**Table 3:** Multivariable associations between the incidence of TB and predictors in the Kyrgyz Republic, (*n* = 24 observations per country, historical period: 2000–2023).

Predictors	Model 1 (Enter Method)		Model 2 (Backward Method)	
	Standardized Coefficients (95% CI (Lower; Upper))	*p*- Value	Standardized Coefficients (95% CI (Lower; Upper))	*p*- Value
Life expectancy at birth, total	−0.213 (−8.234; 4.268)	0.517	-	-
Prevalence of anemia among nonpregnant women	0.652 (−0.169; 12.055)	0.056	0.851 (5.639; 9.877)	<0.001 °
Model parameters	R^2^ = 0.729; overall model *p* < 0.001 °		R^2^ = 0.724; overall model *p* = 0.517	

*° The association was statistically highly significant.*

**Table 4:** Multivariable associations between the incidence of TB and predictors in Tajikistan (*n* = 24 observations per country, historical period: 2000–2023).

Predictors	Model 1 (Enter Method)		Model 2 (Backward Method)	
	Standardized Coefficients (95% CI (Lower; Upper))	*p*- Value	Standardized Coefficients (95% CI (Lower; Upper))	*p*- Value
GDP per capita	−0.470 (−0.102; −0.060)	<0.001 °	−0.476 (−0.101; −0.062)	<0.001 °
Immunization, measles	−0.016 (−1.211; 0.872)	0.737	-	-
Population density	−0.177 (−2.126; −1.710)	0.032 *	−0.179 (−2.108; −0.147)	0.026 *
Prevalence of anemia among nonpregnant women	0.209 (3.633; 16.702)	0.004 **	0.212 (3.988; 16.606)	0.003 **
Prevalence of undernourishment	0.189 (0.137; 1.710)	0.024 *	0.194 (0.194; 1.699)	0.016 *
Model parameters	R^2^ = 0.987; overall model *p* < 0.001 °		R^2^ = 0.999; overall model *p* = 0.737	

* The association was significant. ** The association was highly significant. ° The association was statistically highly significant.

**Table 5:** Multivariable associations between the incidence of TB and predictors in Turkmenistan, (*n* = 24 observations per country, historical period: 2000–2023).

Predictors	Model 1 (Enter Method)		Model 2 (Backward Method)	
	Standardized Coefficients (95% CI (Lower; Upper))	*p*- Value	Standardized Coefficients (95% CI (Lower; Upper))	*p*- Value
Physicians	−0.938 (−205.939; −14.868)	0.026 *	−0.930 (−202.027; −16.911)	0.023 *
Prevalence of anemia among nonpregnant women	1.732 (6.239; 22.358)	0.001 **	1.784 (8.239; 21.227)	<0.001 °
Total greenhouse gas emissions including LULUCF	0.442 (−0.025; 1.732)	0.056	-	-
Model parameters	R^2^ = 0.977; overall model *p* < 0.001 °		R^2^ = 0.973; overall model *p* = 0.843	

* The association was significant ** The association was highly significant. ° The association was statistically highly significant.

**Table 6:** Multivariable associations between the incidence of TB and predictors in Uzbekistan, (*n* = 24 observations per country, historical period: 2000–2023).

Predictors	Model 1 (Enter Method)		Model 2 (Backward method)	
	Standardized Coefficients (95% CI (Lower; Upper))	*p*- Value	Standardized Coefficients (95% CI (Lower; Upper))	*p*- Value
Current health expenditure per capita	−0.110 (−0.278; 0.178)	0.652	-	-
GDP per capita	−0.219 (−0.02; 0.01)	0.503	-	-
Life expectancy at birth, total	−0.590 (−10.342; −0.680)	0.027 *	−0.883 (−10.178;−6.303)	<0.001 °
Model parameters	R^2^ = 0.798; overall model *p* < 0.001 °		R^2^ = 0.780; overall model *p* = 0.213	

* The association was significant. ° The association was statistically highly significant.

## Data Availability

The data presented in this study are provided in the Supplementary Materials.

## Supplementary Materials

The following supporting information can be downloaded at https://www.mdpi.com/article/10.3390/ijerph23010068/s1. Table S1: Description of indicators included in the analysis; Table S2: The list of excluded variables due to unavailability. Table S3: Initial Predictor Set and Final Variables Retained in Country-Specific Models

## Abbreviations

The following abbreviations are used in this manuscript:

95% CI	95% confidence interval
AAPC	Average annual percent change
ARIMA	Autoregressive integrated moving average
BCG	Bacillus Calmette–Guérin
CA	Central Asia
*MTB*	*Mycobacterium tuberculosis*
NAPs	National Action Plans
SDGs	United Nations Sustainable Development Goals
SDHs	Social determinants of health
SPSS	Statistical Package for Social Sciences
TB	Tuberculosis
WB	World Bank
WB DataBank	World Bank DataBank
WHO	World Health Organization
