# Supplementary material for: Impact of Social Determinants of Health on the Incidence of Tuberculosis in Central Asia
Source: Int J Environ Res Public Health. 2026 Jan 1;23(1):68. doi: 10.3390/ijerph23010068 (PMC12840887; doi:10.3390/ijerph23010068)
Supplement: Supplementary file 1 [file ijerph-23-00068-s001.zip › ijerph-3966374-supplementary.pdf]

Supplementary Table S1. Description of indicators included in the analysis

Category	Indicator	Years of data availability
**Air pollution**	Access to clean fuels and technologies for cooking (% of population)	2000-2022
	PM2.5 air pollution, mean annual exposure (micrograms per cubic meter)	2000-2020
	Total greenhouse gas emissions including LULUCF (Mt CO2e)	2000-2022
**Behavior**	Prevalence of HIV, total (% of population ages 15-49)	2000-2022 (for Kyrgyz Republic and Tajikistan)
	Incidence of HIV, all (per 1,000 uninfected population)	2000-2022 (Kyrgyz Republic and Tajikistan)
	Total alcohol consumption per capita (liters of pure alcohol, projected estimates, 15+ years of age)	2000-2020
**Economic development and equity**	GDP per capita (current US$)	2000-2023
	Gini index	2001-2021 (Kazakhstan only), 2000-2022 (Kyrgyz Republic only)
	Inflation, consumer prices (annual %)	2000-2023 (Kazakhstan and Kyrgyz Republic)
**Education**	Compulsory education, duration (years)	2000-2023
**Employment**	Unemployment, total (% of total labor force) (modeled ILO estimate)	2000-2023
**Health and healthcare services**	Births attended by skilled health staff (% of total)	2000-2020, 2022 (Uzbekistan only)
	Immunization, measles (% of children ages 12-23 months)	2000-2023
	Physicians (per 1,000 people)	2000-2020 (Kazakhstan only), 2000-2021 (for Tajikistan, Turkmenistan, and Uzbekistan)
	Prevalence of anemia among non-pregnant women (% of women ages 15-49)	2000-2023
	Prevalence of anemia among pregnant women (%)	2000-2023
	Prevalence of undernourishment (% of population)	2000-2022 (Kazakhstan only), 2001-2022 (for Kyrgyz Republic, Tajikistan, Turkmenistan, and Uzbekistan)
	Tuberculosis case detection rate (%, all forms)	2000-2023 (Kazakhstan and Uzbekistan), 2000-2023 (Kyrgyz Republic, Tajikistan, and Turkmenistan
	Tuberculosis treatment success rate (% of new cases)	2000-2022 (Kazakhstan, Kyrgyz Republic, Tajikistan, and Uzbekistan), 2000-2021 (Turkmenistan only)
	Vitamin A supplementation coverage rate (% of children ages 6-59 months)	2000-2022 (Tajikistan only)
**Health Expenditure**	Current health expenditure per capita (current	2000-2022

	US$)	
**Population**	Birth rate, crude (per 1,000 people)	2000-2023
	Death rate, crude (per 1,000 people)	2000-2023
	Life expectancy at birth, total (years)	2000-2023
	Population ages 0-14 (% of total population)	2000-2023
	Population ages 65 and above (% of total population)	2000-2023
	Population density (people per sq. km of land area)	2000-2022
	Population growth (annual %)	2000-2023
	Rural population (% of total population)	2000-2023

Supplementary Tables S2. The list of excluded variables due to unavailability

**Indicators**	**Data availability**
Adequacy of social protection and labor programs (% of total welfare of beneficiary households)	Separate years only, depending on country (NA for Turkmenistan)
ARI treatment (% of children under 5 taken to a health provider)	Separate years only, depending on country
Benefit incidence of social insurance programs to poorest quintile (% of total social insurance benefits)	Separate years only, depending on country (NA for Turkmenistan)
Borrowers from commercial banks (per 1,000 adults)	Separate years only, depending on country (NA for Turkmenistan)
Cause of death, by communicable diseases and maternal, prenatal and nutrition conditions (% of total)	Separate years only, depending on country
Cause of death, by non-communicable diseases (% of total)	Separate years only, depending on country
Community health workers (per 1,000 people)	Separate years only for Uzbekistan (NA for Kazakhstan, Kyrgyz Republic, Tajikistan, and Turkmenistan)
Coverage of social insurance programs (% of population)	Separate years only, depending on country (NA for Turkmenistan)
Coverage of social insurance programs in 3rd quintile (% of population)	Separate years only, depending on country (NA for Turkmenistan)
Diabetes prevalence (% of population ages 20 to 79)	Separate years only, depending on country
Educational attainment, at least completed primary, population 25+ years, total (%) (cumulative)	Separate years only, depending on country
Educational attainment, at least completed upper secondary, population 25+, total (%) (cumulative)	Separate years only, depending on country
Educational attainment, at least Bachelor's or equivalent, population 25+, total (%) (cumulative)	Separate years only, depending on country
Educational attainment, at least Master's or equivalent, population 25+, total (%) (cumulative)	Separate years only, depending on country (NA for Turkmenistan and Uzbekistan)
Exclusive breastfeeding (% of children under 6 months)	Separate years only, depending on country
Human capital index (HCI) (scale 0-1)	Separate years only, depending on country (NA for Turkmenistan)
Intentional homicides (per 100,000 people)	Separate years only, depending on country
International migrant stock (% of population)	Separate years only, depending on country
Mortality rate attributed to household and ambient air pollution, age-standardized (per 100,000 population)	Separate years only, depending on country

Multidimensional poverty headcount ratio (UNDP) (% of population)	Separate years only, depending on country
PM2.5 air pollution, population exposed to levels exceeding WHO guideline value (% of total)	Separate years only, depending on country
Population living in slums (% of urban population)	Separate years only, depending on country
Prevalence of anemia among children (% of children ages 6-59 months)	Separate years only, depending on country
Prevalence of current tobacco use (% of adults)	Separate years only, depending on country (NA for Tajikistan)
Prevalence of moderate or severe food insecurity in the population (%)	Separate years only, depending on country (NA for Turkmenistan and Uzbekistan)
Prevalence of severe food insecurity in the population (%)	Separate years only, depending on country (NA for Turkmenistan and Uzbekistan)
Prevalence of severe food insecurity in the population (%)	Separate years only, depending on country (NA for Turkmenistan and Uzbekistan)
Proportion of population pushed below the $3.65 ($ 2017 PPP) poverty line by out-of-pocket health care expenditure (%)	Separate years only, depending on country (NA for Turkmenistan)
Proportion of population pushed further below the $2.15 ($ 2017 PPP) poverty line by out-of-pocket health care expenditure (%)	Separate years only, depending on country (NA for Turkmenistan)
Proportion of population spending more than 10% of household consumption or income on out-of-pocket health care expenditure (%)	Separate years only, depending on country (NA for Turkmenistan)
School enrollment, secondary (% net)	Separate years only, depending on country (NA for Turkmenistan)
UHC service coverage index	Separate years only, depending on country

Supplementary Table S3. Initial Predictor Set and Final Variables Retained in Country-Specific Models

Initial Set	Correlation coefficient*	Final Set	Enter Method Standardized coefficients (95% Confidence Interval); p-value	Backward Model Standardized coefficients (95% Confidence Interval); p-value
**Kazakhstan**				
Access to clean fuels and technologies for cooking	-0.855	Access to clean fuels and technologies for cooking	-0.644 (-16.779; -5.725); <0.001	-0.777 (-16.222; -10.922); <0.001
Death rate, crude	0.892	-	-	-
Life expectancy at birth, total	-0.961	Life expectancy at birth, total	-0.103 (-4.541; 1.660); 0.328	-
Population ages 0-14	-0.834	Population ages 0-14	-0.638 (-24.859; -11.706); <0.001	-0.733 (-24.338; -17.639); <0.001
Population density	-0.928	Population density	0.298 (3.356; 52.930); 0.028	0.386 (18.961; 54.039); <0.001
Population growth	-0.892	-	-	-
Prevalence of undernourishment	0.865	Prevalence of undernourishment	0.098 (0.042; 5.314); 0.047	0.085 (-0.199; 4.837)
Rural population	0.921	-	-	-
Unemployment, total	0.814	-	-	-
**Kyrgyz Republic**				
Life expectancy at birth, total	-0.823	Life expectancy at birth, total	-0.213 (-8.234; 4.268); 0.517	-
Population density	-0.824	-	-	-
Prevalence of anemia among nonpregnant woman	0.851	Prevalence of anemia among nonpregnant women	0.652 (-0.169; 12.055); 0.056	-
**Tajikistan**				
Access to clean fuels and technologies for cooking	-0.954	-	-	-
Current health expenditure per capita	-0.949	-	-	-
Death rate, crude	0.896	-	-	-
GDP per capita	-0.950	GDP per capita	-0.470 (-0.102; -0.060); <0.001	-0.476 (-0.101; -0.062); <0.001
Immunization, measles	-0.827	Immunization, measles	-0.016 (-1.211;0.872); 0.737	-
Incidence of HIV, all	-0.964	-	-	-
Life expectancy at birth, total	-0.940	-	-	-

Population ages 0-14	0.909	-	-	-
Population density	-0.906	Population density	-0.177 (-2.126; -1.710); 0.032	-0.179 (-2.108; -0.147); 0.026
Prevalence of anemia among nonpregnant woman	0.934	Prevalence of anemia among nonpregnant women	0.209 (3.633; 16.702); 0.004	0.212 (3.988; 16.606); 0.003
Prevalence of undernourishment	0.903	Prevalence of undernourishment	0.189 (0.137; 1.710); 0.024	0.194 (0.194; 1.699); 0.016
		**Turkmenistan**		
Current health expenditure per capita	-0.832	-	-	-
Death rate, crude	0.848	-	-	-
GDP per capita	-0.899	-	-	-
Life expectancy at birth, total	-0.913	-	-	-
Physicians	0.802	Physicians	-0.938 (-205.939; -14.868); 0.026	-0.930 (-202.027; -16.911); 0.023
Population density	-0.919	-	-	-
Prevalence of anemia among nonpregnant woman	0.881	Prevalence of anemia among nonpregnant women	1.732 (6.239;22.358); <0.001	1.784 (8.239; 21.227); <0.001
Rural population	0.915	-	-	-
Total greenhouse gas emissions including LULUCF	-0.841	Total greenhouse gas emissions including LULUCF	0.442 (-0.025; 1.732); 0.056	-
Unemployment, total	0.819	-	-	-
		**Uzbekistan**		
Birth rate, crude	-0.927	-	-	-
Current health expenditure per capita	-0.805	Current health expenditure per capita	-0.110 (-0.278; 0.178); 0.652	-
GDP per capita	-0.858	GDP per capita	-0.219 (-0.02; 0.01); 0.503	-
Life expectancy at birth, total	-0.883	Life expectancy at birth, total	-0.590 (-10.342; -0.680); 0.027	-0.883 (-10.178;-6.303); <0.001
Population density	-0.890	-	-	-
Population growth	-0.945	-	-	-
Prevalence of anemia among nonpregnant woman	0.863	-	-	-
Prevalence of anemia among pregnant women	0.815	-	-	-

*all p-values<0.001
